# Prevalence of Pulmonary Arterial Hypertension among Sickle Cell Disease Patients in AL Hassa

**DOI:** 10.5539/gjhs.v5n5p174

**Published:** 2013-06-30

**Authors:** Emad Ali Saleh AL-Khoufi

**Affiliations:** 1 College of Medicine, King Faisal University, Hofuf, Saudi Arabia

**Keywords:** sickle cell disease, pulmonary arterial hypertension, prevalence, Saudi Arabia

## Abstract

**Background:**

The prevalence of pulmonary arterial hypertension (PAH) in Saudi adults with sickle cell disease (SCD), the mechanism of its development, and its prospective prognostic significance are unknown.

**Objective:**

To assess the prevalence of PAH among sickle cell disease patients attended hematology outpatient clinic at King Fahad Hospital, Al Hassa, Saudi Arabia.

**Methods:**

Doppler echocardiography was performed for assessments of pulmonary- arterial systolic pressure (PASP) on 203 consecutive patients (102 men and 101 women) aged > 11 years, attending hematology clinic at King Fahad Hospital, Al Hassa, Saudi Arabia. Pulmonary hypertension was prospectively defined as a tricuspid regurgitant jet velocity (TRJV) of at least 2.5 m per second which can be estimate PASP equal or more than 25 mmHg.

**Results:**

Doppler-defined pulmonary arterial hypertension was diagnosed in 37.1% among 202 patients included in study (after one female patient was excluded) using a cutoff of PASP ≥25 mmHg.

**Conclusion:**

The prevalence of PAH among adults Saudis with SCD is higher than that reported from the developed countries. Further assessment using invasive techniques is required coupled employing analytical study design to predict the factors that favor the development of PAH among Saudi patients are required.

## 1. Background

Sickle cell anemia is an autosomal recessive disorder causing production of abnormal β globin chains, an amino acid substitution in the gene coding for the β chain resulting in the production of HbS rather than HbA. HbA2 and HbF are still produced. The homozygote (SS) has sickle-cell anemia (HbSS), and heterozygotes (HbAS) have sickle-cell trait (Bender & Williams 2012). Sickle cell disease (SCD) is a health problem in Saudi Arabia, especially in Southern, Western, and Eastern regions where the genes frequency responsible of this disease is quite prevalent with a range of 0.15-0.25. In Al Hassa Governorate, the prevalence of homozygote SCD prevalence ranges from1 to1.5% (Alabdulaali, 2007). Pulmonary arterial hypertension (PAH), is a syndrome characterized by increased pulmonary vascular resistance and remodeling, and is associated with significant morbidity and mortality, which are directly related to cardiac function (Fisher et al., 2009). PAH affects approximately 6%-35% of adults with SCD (Gladwin et al., 2004; Ataga et al., 2006; Parent et al., 2011). In SCD, PAH has been defined by an elevated tricuspid regurgitant jet velocity (TRJV) on trans-thoracic echocardiography (TTE). However, subsequent studies using direct measurement of PASP by right heart catheterization indicate an overestimation of PAH by Trans-thoracic echocardiography (TTE) (Parent et al., 2011). PAH is associated with markedly increased mortality (Gladwin et al., 2004; Ataga et al., 2006; De Castro et al., 2008). Some individuals are relatively asymptomatic in the early stages of PAH. PAH is a serious complication of SCD in adults and accounted to affect 32% of adult patients. There are limited data on the prevalence of PAH among adults in Al Hassa region, Saudi Arabia. Factors and patients’ characteristics are largely unknown. The definitive diagnosis of PAH is currently established through right-heart catheterization; however, this is an invasive technique and is not suitable for screening. Accurate noninvasive assessment of pulmonary arterial pressure is desirable both for diagnostic purposes and to assess response to therapy (Pashankar et al., 2008). Trans-thoracic Doppler echocardiography is recommended as the initial noninvasive modality in the screening and evaluation of PAH. Doppler-echocardiogram can be used to measure the velocity of regurgitant blood across the tricuspid valve using the modified Bernoulli's equation to calculate the PASP (Chaudhry et al., 2011). Estimates of PAH by Doppler echocardiography correlate well with those obtained by cardiac catheterization, moreover, Doppler echocardiography is safe and accurate method to detect PAH especially in children (Colombatti et al 201). This study aimed to assess the prevalence of PAH among SCD patients attended hematology outpatient clinic at King Fahad Hospital, Al Hassa, Saudi Arabia.

## 2. Patients and Methods

### 2.1 Setting and Design

This study included all adults patients with SCD of diverse genetic types including (HbSS, HbSC, HbS/β thalassaemia) attended and admitted to King Fahad Hospital located in Al Hassa, Eastern Province, Kingdom of Saudi Arabia.

### 2.2 Data Collection: The Following Tools Were Used for Data Collection

2.2.1 Reviewing of patients medical records available at King Fahad Hospital over a one year period. King Fahad Hospital is a secondary level of care hospital located in Hofuf and serving about one million populations. All patients with SCD (aged > 11 years) are receiving their routine care and management in this hospital through the Hematology clinics and inpatient care during their admission. All patients records were revised using a pre-tested data compilation form to gather information regarding: socio-demographics including age, gender, residence and educational/occupational statuses. Age at diagnosis with SCD symptoms, current management procedures, complications (history and their nature), admission frequency and reasons for these admissions, previous pulmonary problems, intensive care and reasons/frequency, history of pulmonary syndrome. The presence of other comorbid disease conditions (diabetes/bronchial asthma etc.). Laboratory investigations including the disease genotype/fetal hemoglobin, serum ferritin level and other hematological parameters. The results of previous echocardiogram (if any).

End points:

The primary end point was detecting PAH,
Secondary points include frequency of admission, acute chest syndrome,
Intensive care unit (ICU) admission and mortality related to PAH.

Exclusion criteria: all cases with secondary cause of PAH including:

SCD patients with congenital heart disease or any obstructive heart lesions, collagen vascular disease, HIV, Schistosomiasis, PAH associated with lung disease and or persistent drop in oxygen levels (hypoxia) including; chronic obstructive lung disease (COPD), sleep apnea, pulmonary fibrosis and living at high elevation and pulmonary embolism.

2.2.2 Eligible patients with valid and complete records and with regular compliance and follow up at the hematology clinics were referred after obtaining their (or their legal) guardians’ written consent form for Echocardiogram for the diagnosis of PAH.

2.2.3 All patients underwent comprehensive physical examination and thorough history taking followed by Echocardiography to determine TRJV and to calculate PASP. Indices of hemolysis and a laboratory study were also collected to detect varies risk factors possible for the development of PAH.

### 2.3 Data Management and Statistical Analysis

Data entered and analyzed using SPSS version 16.0 (SPSS Inc. Chicago, IL). For categorical data, proportions, frequency and percentage will be used for expression; Chi square and *Z* test for proportions were used for comparison. Continuous data were expressed using median, mean and standard deviation. Intercorrelation matrix was generated with reporting of correlation coefficient to determine the possible risk factors for the development of PAH in SCD patients. *P* value < 0.05 considered statistically significant.

### 2.4 Ethical Considerations

Permissions were obtained from King Faisal University as well as from the authorities of King Fahad hospital after approval of the study protocol and data complication form. Written consent forms were obtained from the included patients/their guardians after receiving proper orientations regarding the study objectives and outcome. Data confidentiality was maintained all through the study.

## 3. Results

Table 1 displays the demographic and clinical characteristics of the included patients with SCD. A total of 202 patients were included females constituted 49.5% (n=100). The age range for the whole sample was 11-74 years (mean=28.80±11.60), females were younger than males but without statistical significance. The table also shows the total hemoglobin level, hemoglobin S; the concentration of total Hb was significantly higher among males (P=0.001).

**Table 1:** Characteristics of the included sickle cell disease patients, King Fahad Hospital

Characteristics	Gender: No. (%)		P value

Males (N=102)	Females (N=100)		
Age (in years):			
Range	12-67	11-74	
Median (mean ±SD)	27.0(28.10±10.97)	26.0(29.45±12.2)	0.409*
**Hemoglobin levels:** Median (mean± SD)			
Hemoglobin total	9.5(9.40±1.73)	8.8(8.7±1.20)	0.001*
Hemoglobin A1	0.0(7.45±11.79)	0.0(6.64±10.48)	0.607*
Hemoglobin S	75.50(74.80±9.98)	75.2(73.64±9.91)	0.399^*^
**Pulmonary arterial hypertension:**			
Yes	41(40.2)	34(34.0)	
No	61(59.8)	66(66.0)	0.562**
**SPAP mmHg:** mean± SD	33.05±7.56	32.41±7.23	0.539^*^
**Acute chest syndrome:**			
Yes	25(24.5)	23(23.0)	
No	77(75.5)	77(77.0)	0.800**
**Intensive care unit (ICU) admission:**			
Yes	52(51.0)	38(38.0)	
No	50(49.0)	62(62.0)	0.060**
**Frequency of hospital admission:**			
1-2 times	14(26.9)	8(21.1)	
Three times	23(44.2)	14(36.8)	
≥ 4 time	15(28.8)	16(42.1)	0.031**
**Cause of ICU admission:**			
1-Pulmonary embolism	1(1.0)	- -	
2-Vaso-occlusive crisis	9(8.8)	1(1.0)	--
3-Acute chest syndrome	21(20.6)	19(19.0)	--
4-Low hemoglobin	15(14.7)	12(12.0)	--
5-Post operative	1(1.0)	1(1.0)	--
6-Sepsis	1(1.0)	2(2.0)	--
7-Chest infections	3(2.9)	2(2.0)	--
8-Stroke (ischemic)	1(1.0)	--	--
9-Internal hemorrhage	--	1(1.0)	- -
**Mortality: no. (%)**	4(3.9)	4(4.0)	--
**Causes of death:**			
Hemolytic crisis	1(1.0)	--	--
Internal hemorrhage	- -	1(1.0)	--
Septic shock	2(2.0)	3(3.0)	--
Multi-organs failure	1(1.0)	--	--

* t-test for independent samples.

** Chi square test.

Those with PAH accounted for 37.1% (n=75), more among males but without statistical significance. SPAP showed a mean of 32.7±7.40 mmHg. Those developed acute chest syndrome constituted 23.8 % (n=48), ICU admission was recorded in 90 patients (44.6%) more among males (51.0% vs. 38.0% in females). The frequency of hospital admission also shows no significant difference in relation to gender. The most frequently encountered reason for admission included acute chest syndrome and low hemoglobin concentrations. Overall mortality was 4.0 % and almost 90% of the deaths were due to septic shock.

Table 2 demonstrates the distribution of the different demographic and clinical characteristics in relation to the development of acute chest syndrome.

**Table 2:** Acute chest syndrome in relation to demographic and clinical characteristics of the included patients

P value	Acute chest syndrome: No. (%)		Characteristics
	Present (N=48)	Absent (N=154)	
**Gender:**			0.800*
Males	77(50.0)	25(52.1)	
Females	77(50.0)	23(47.9)	
**Age (in years): mean ±SD**	28.28±11.75	30.33±11.14	0.286**
**Hemoglobin levels: mean± SD**			
Hemoglobin total	9.01±1.45	9.21±1.73	0.427**
Hemoglobin A1	6.64±10.58	8.39±12.83	0.343**
Hemoglobin S	74.78±9.47	72.49±9.94	0.149**
**Pulmonary arterial hypertension:**			0.152*
Yes	47(30.5)	20(41.7)	
No	107(69.5)	28(58.3)	
**SPAP mmHg: mean± SD**	31.36±6.72	35.11±7.94	0.001**
**Intensive care unit (ICU) admission:**			0.001*
Yes	48(31.2)	42(87.5)	
No	106(68.8)	6(12.5)	
**Frequency of hospital admission:**			0.105*
1-2 times	13(27.1)	9(18.8)	
Three times	24(50.0)	23(47.9)	
≥ 4 time	11(22.9)	16(33.3)	
**Cause of hospital admission:**			
Pulmonary embolism	1(0.6)	--	--
Vaso-Occlusive crisis	7(4.5)	3(6.3)	--
Acute chest syndrome	3(1.9)	37(77.1)	--
Low hemoglobin	26(16.9)	1(2.1)	--
Post operative	2(1.3)	--	--
Sepsis	3(1.9)	--	--
Chest infections	5(3.2)	--	--
Stroke (ischemic)	1(0.6)	--	--
Internal hemorrhage	--	1(2.1)	--
**Mortality: no. (%)**	1(0.6)	7(14.6)	--
Causes of death:			
Hemolytic crisis	--	1(2.1)	--
Internal hemorrhage	--	1(2.1)	--
Septic shock	1(0.6)	4(8.3)	--
Multi-organ failure	--	1(2.1)	--

* Chi square test,

** t-test for independent samples.

Acute chest syndrome was encountered more among male patients, of relatively older age, and with presence of PAH (significantly higher among those with SPAP) and with frequent hospital admission. Mortality was significantly higher among patients with acute chest syndrome.

Table 3 demonstrates the inter correlation matrix for the possible correlates of arterial pulmonary hypertension in sickle cell patients. Older age, the occurrence of acute chest syndrome and frequency of hospital admissions were all positively and significantly correlated with the development of PAH.

**Table 3:** Intercorrelation matrix for the possible correlates of PAH among SCD patients, King Fahad Hospital in Al Hassa

Variables	Sex	Pulmonary arterial hypertension	SPAP mmHg	ICU Admission	Acute chest syndrome	Frequency of Hospital admission	Hemoglobin	Hemoglobin A1	Hemoglobin S
Age (in years)	.067	.290(**)	.158	.182(*)	.090	.049	-.179(*)	-.008	-.017
Sex		-.057	.040	-.055	-.009	-.124	-.228(**)	-.039	-.055
Pulmonary arterial hypertension				-.133	.253(**)	.428(**)	-.077	-.006	.010
SPAP mmHg				-.143	.284(*)	.179	.002 0.988	-.080 0.489	.054 0.642
ICU Admission					-.146(*)	-.385(**)	.061	-.104	.015
Acute chest syndrome						.487(**)	.053	.061	-.089
Frequency hospital admissions							-.002	.124	-.070
Hemoglobin								-.111	.115
Hemoglobin A1									-.762(**)

* Correlation is significant at the 0.05 level (2-tailed).

** Correlation is significant at the 0.01 level (2-tailed).

## 4. Discussion

An initial study in Howard University, USA, using echocardiographic assessment of TRJV ≥2.5m/sec as diagnostic criteria, demonstrated PAH in 32% of adult sickle-cell patients, and the prevalence appeared to increase with age of the patients (Parent et al., 2011). In patients between 40 and 49 years old, the prevalence was 40% and increased to 55–60% by age 50 and above. Other studies have documented prevalence rates between 20 and 40% (Sutton et al., 1994; Simmons et al., 1988). Sickle-cell anemia patients with PAH have a significantly increased mortality rate compared with patients without pulmonary arterial hypertension. Sutton and colleagues (1994) reported 40% mortality rate in sickle-cell patients with PAH at 22 months after diagnosis (Odd ratio 7.86; 95% confidence interval = 2.63–23.4) compared with sickle-cell patients without pulmonary arterial hypertension. Castro et al. (2003) in a study of 34 adult sickle-cell patients who underwent right heart catheterization for evaluation of PAH found increased pulmonary arterial pressure in 58.8% on initial catheterization. During 23–45 months of follow up, 11 of these 20 (55.0%) died compared with 3 of the 14 without PAH (21.0%). In this study among adult patients SCD, the prevalence of a PAH defined as TRJV of at least 2.5 m per second on echocardiography was 37.1% (n=75), more among males, relatively of older age and with higher hemoglobin S concentration, and frequent ICU admission and with higher PASP. Similar study by Gladwin et al. (2004) have reported close prevalence in 2004, they performed Doppler echocardiography assessments of PASP in 195 consecutive patients (82 men and 113 women) and the result by Doppler-defined PAH have reported a prevalence of 32%. The rate found in this study was lower and inconsistent with that reported from a French study where all patients with SCD underwent Doppler echocardiography in a referral center, with measurement of TRJV and rate consistent with those in other prospective studies at referral centers in France (Fonseca et al., 2012). In the previous study, the prevalence of PAH by echocardiography was 27% (n=109) of the 398 patient included, the difference may be due to use of invasive method where the prevalence of PAH was confirmed on right heart catheterization, they found that only 6% (n=24) of the included 96 patient have had TRJV with at least 2.5m/sec. Colombatti et al. (2010) have found that the positive predictive value of echocardiography for the detection of PAH was 25%.

Consistent with our results, one study has found similar predictors for mortality among SCD with confirmed PAH where mortality was associated with frequent vaso-occlusive crisis and acute chest syndrome (Parent et al., 2011). Contrary to our results, the previous study found that the hemoglobin level had no role in the development of PAH. In contrast, they observed that in patients with confirmed PAH there was a significantly increased lactate dehydrogenase (LDH) and aspartate aminotransferase (AST) levels, which may also be influenced by liver dysfunction. Compared to studies from the Western countries, our results revealed higher prevalence of PAH and this may be due to the higher prevalence of the disease in Kingdom of Saudi Arabia in Al Hassa region and the unavailability of proper management and early detection of PAH.

## 5. Conclusion

The prevalence of PAH among adults Saudis with SCD is higher than that reported from the developed countries. Further assessment using invasive techniques is required coupled employing analytical study design to predict the factors that favor the development of PAH among Saudi patients are required.
